# Clinical and pathological characteristics of patients with pulmonary inflammatory pseudotumors: An 18-year retrospective study of 31 cases

**DOI:** 10.1097/MD.0000000000027040

**Published:** 2021-09-03

**Authors:** Heng-Chi Chen, Qiang Fu, Yan Song, Da-Li Wang

**Affiliations:** a Department of Thoracic Surgery, National Cancer Center/National Clinical Research Center for Cancer/Cancer Hospital, Chinese Academy of Medical Sciences & Peking Union Medical College, Beijing, China; b Department of Pathology, National Cancer Center/National Clinical Research Center for Cancer/Cancer Hospital, Chinese Academy of Medical Sciences & Peking Union Medical College, Beijing, China.

**Keywords:** clinical characteristics, pathological characteristics, pulmonary inflammatory pseudotumors

## Abstract

To investigate the clinical and pathological characteristics in patients with pulmonary inflammatory pseudotumors (PIP).

This retrospective study included 31 patients with PIP from 2001 to 2019. Preoperative computed tomography scan was performed in all patients. Clinical and pathological characteristics were collected and analyzed.

Thirty-one patients (16 female and 15 male) were recruited, with a median age of 57 years (range, 11–72 years). Eight (25.8%) patients were asymptomatic, and the others had symptoms characterized by cough with sputum, chest and back pain, dry cough, fever and blood in sputum, or hemoptysis. All cases were single lesions, including 23 cases in the right lung, and 8 cases in the left lung. Computed tomography scan demonstrated irregular lobulated nodules or masses in 14 patients, and regular round or oval nodules or masses in 11 cases. The blurred edge of tumors and spiculation was found in 12 cases. Microscopic results were characterized by the collection of inflammatory mesenchymal cells. Immunohistochemical examination showed vimentin, smooth muscle actin, and anaplastic lymphoma kinase positive. Complete tumor resection was obtained in all cases. No recurrence or metastasis was observed during the follow-up period.

PIP has a variety of manifestations. Preoperative diagnosis is difficult to reach. The final diagnosis still depends on the pathological and immunohistochemical examination. Complete surgical resection is the main treatment at present, and the overall prognosis is good.

## 1 Introduction

Inflammatory pseudotumors, also known as inflammatory myofibroblastic tumor, are rare nonmalignant lesions.^[1]^ It can occur in the soft tissues of almost all organs, of which the lung is the most common.^[2]^ Pulmonary inflammatory pseudotumors (PIP) is characterized by an irregular proliferation of myofibroblasts accompanied by an infiltrate of inflammatory cells.^[3]^ The actual incidence of PIP is unknown but it is estimated to be between 0.04% and 1.2%.^[4]^ Diagnosis of this tumor is difficult without surgical resection or biopsy.^[5–7]^

Due to its rarity, the etiology, pathogenesis, and response to treatment of PIP are unclear. In addition, the institutional experience is usually limited and there are few reports in the literature.^[1,7,8]^ The few series in the literature have showed that the clinical behavior of the patients vary greatly. In order to better understand the clinical significance and treatment standards of PIP, we retrospectively analyzed 31 cases with PIP to ascertain their clinical and pathological characteristics.

## 2 Materials and methods

### 2.1 Patients

We retrospectively reviewed 31 patients with PIP who underwent surgical treatment in our hospital between 2001 and 2019. The inclusion criteria were as follows: patients with PIP diagnosed by postoperative histopathology; and patients underwent surgical treatment in our hospital. The exclusion criteria were as follows: patients with severe cardiovascular and cerebrovascular diseases, liver or kidney dysfunction etc; patients with mental disorders; and pregnant women. This study was approved by the ethics committee of National Cancer Center/National Clinical Research Center for Cancer/Cancer Hospital. Written informed consent was obtained from all participants and their guardians.

### 2.2 Therapy and follow-up

Preoperative thoracic computed tomography (CT) scans was performed in all cases, including 25 cases in our hospital and 6 cases in other hospitals. Other preoperative examinations included an abdominal ultrasound and bronchoscopy. All cases underwent surgery treatment either by thoracotomy or by video assisted thoracoscopy. An intraoperative frozen section histology study was performed in all cases. For a definitive histology study, the surgical specimens were stained with hematoxylin and eosin and then subjected to conventional histology. Vimentin, smooth muscle actin, and anaplastic lymphoma kinase were detected by immunohistochemical analysis.

The demographic and clinic characteristics were collected from the medical record of each case, including gender, age, smoking history, symptoms, family history, imaging findings, pathological findings, operation, and outcome. The patients were followed up by inquiring the outpatient records or telephone calls. The patients were followed up to July 2020, with a median follow-up of 95 months (range, 21–144 months).

## 3 Results

### 3.1 Baseline characteristics

A total of 31 patients (15 males, 16 females; median age: 57 years; range, 11–72 years) with PIP were enrolled in this retrospective study from 2001 to 2019. Among the 31 patients, 10 (32.2%) had a history of smoking and 7 (22.6%) patients had a clear family history of tumors. The baseline characteristics of included patients were showed in Table 1. Eight (25.8%) patients were asymptomatic and lung masses were occasionally detected during a routine examination. The common symptoms were cough with sputum (54.8%) and blood in sputum or hemoptysis (45.2%).

**Table 1:** The baseline characteristics of included patients.

Characteristic	Number (n, %)
Age (yr), median/range	57 (11–72)
Smoking history	10 (32.2)
Family history of tumors	7 (22.6)
Lung cancer	3 (9.7)
Breast cancer	2 (6.5)
Lung cancer complicated with kidney cancer	1 (3.2)
Unknown	1 (3.2)
Gender	
Male	15 (48.4)
Female	16 (51.6)
Symptom	
Asymptomatic	8 (25.8)
Cough with sputum	17 (54.8)
Blood in sputum or hemoptysis	14 (45.2)
Chest and back pain	3 (9.7)
Dry cough	2 (6.5)
Fever	2 (6.5)

### 3.2 Imaging findings

The CT scan showed that all cases were single lesions, which located on the left upper lobe (9.7%), left lower lobe (16.1%), right upper lobe (25.8%), right lower lobe (29.0%), right middle lobe (12.9%) or right middle bronchus (6.5%).

**Figure 1:**
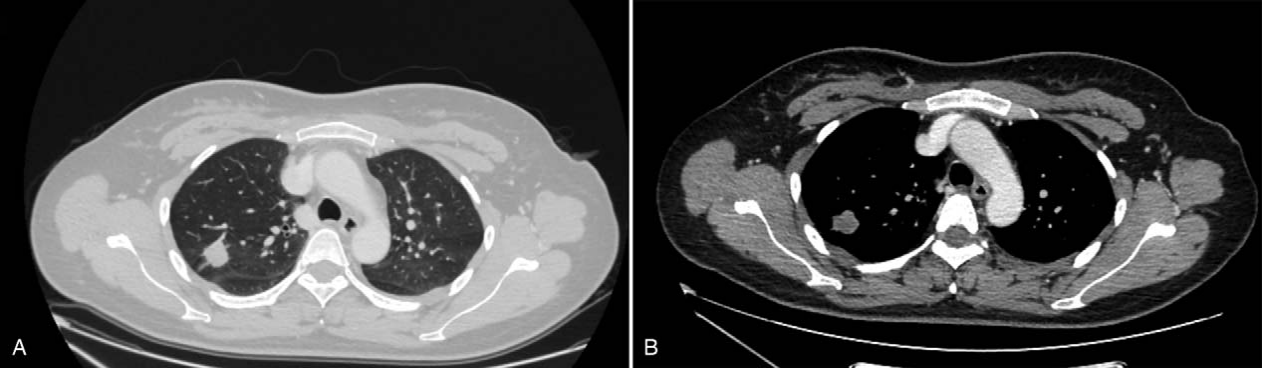
Computed tomography in patients with pulmonary inflammatory pseudotumor. Lung windows (A) and mediastinal windows (B) showing a right upper lobe tumor with speculation.

The CT images were obtained from only 25 cases. The CT scan demonstrated irregular lobulated nodules or masses in 14 patients, and regular round or oval nodules or masses in 11 cases. Intraluminal mass was found in 3 cases, in which 1 was located in the left upper lobar bronchus and 2 were located in the right middle bronchus. The blurred edge of tumors and spiculation was found in 12 cases (Fig. 1), and calcifications were seen in 2 cases.

Through preoperative CT examination, 13 cases were considered as lung cancer or other malignant tumors, 3 cases were inflammation, 1 case was tuberculosis, 1 case was sclerosing alveolar cell tumor, and 1 case was benign pulmonary nodule. Four cases could not be identified as benign or malignant tumors, and only 1 case was considered as PIP. In addition, 1 case was considered as thymoma preoperatively because the tumor was large and protruded into the mediastinum.

### 3.3 Pathological characteristics

**Figure 2:**
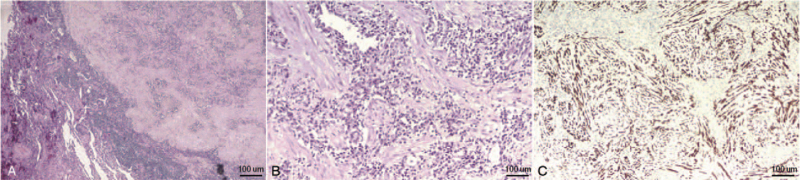
Pathological characteristics of pulmonary inflammatory pseudotumor. A, Microscopic findings of the biopsy specimen showed dense lymphoplasmacytic infiltration within fibrotic stroma (H&E stain at 40×). B, Microscopic findings of the biopsy specimen showed proliferation of myofibroblastic spindle cells intermixed with an inflammatory infiltrate of plasma cells and lymphocytes (H&E stain at 200×). C, Immunohistochemistry examination showed that the spindle cells were positive for anaplastic lymphoma kinase.

The gross specimen showed that the tumor was a solid nodule or mass, and the section was grayish-white, grayish-brown, or grayish-yellow. Multiple small abscesses were found around the tumor in 5 cases. Microscopic results were characterized by the collection of inflammatory mesenchymal cells (lymphocytes, plasma cells, and spindle cells) (Fig. 2A and 2B). Eight patients in our hospital underwent pathological immunohistochemical examination, of which 7 were positive for vimentin, 5 were positive for smooth muscle actin, and 1 was positive for anaplastic lymphoma kinase (Fig. 2C). Of the 31 cases, 18 received lymph node sampling or dissection, and one of them had lymph node metastasis.

### 3.4 Therapy and follow-up

All cases underwent surgery treatment, including lobectomy (67.7%), wedge resection (22.6%), combined lobectomy (6.5%), and pneumonectomy (3.2%). Complete tumor resection was obtained in all cases. The tumor lesion diameter was ranged from 0.7 to 11 cm. No obvious complications occurred after surgery. In addition, none of the cases received chemotherapy and radiotherapy after surgery.

Of the 31 cases, 24 were followed up. The cases were followed up to July 2020, with a median follow-up of 95 months (range, 21–144 months). There was no recurrence or metastasis.

## 4 Discussion

PIP is rare, which was first reported in 1939.^[9]^ The incidence rate of PIP is reported to be 0.04% to 1% of all lung tumors.^[10]^ Initially, it is generally considered to be a non-neoplastic reactive inflammation.^[11]^ With the development of research, PIP is considered to be a benign tumor with a histologic feature of spindle cell proliferation accompanied by obvious inflammatory cell infiltration.^[12]^ However, some evidences showed that PIP has the particular potential for invasion and recurrence.^[13,14]^ At present, the exact etiology and pathogenesis of PIP are still unclear. In this study, we retrospectively analyzed 31 cases with PIP to explore their clinical and pathological characteristics.

PIP has a variety of clinical manifestations. Previous study showed that patients with PIP may remain asymptomatic in 30% to 70% of cases.^[15]^ When symptoms occur they are represented by fever, cough, chest pain, hemoptysis, weight loss, or respiratory infections.^[16]^ In this study, we found that 25.8% patients were asymptomatic, and the common symptoms were cough with sputum (54.8%) and blood in sputum or hemoptysis (45.2%). This disease has no particular gender predilection, and the mean ages of patients ranged from 27 to 50 years.^[4,5]^ In this study, 51.6% of patients were female, and the median age of 57 in our series was slightly older than that given in other reports. The probable reason was that a certain proportion of patients with PIP exhibited no clinical symptom, and the lesions were usually detected by chance on chest radiographs.

According to the literature, PIP was mostly found in the lower lobe of the lung, and enhanced scan showed moderate to high contrast enhancement in the arterial and venous phase.^[17,18]^ In our study, tumors of 20 cases were located in the middle or lower lobe, which was basically consistent with the existing reports. However, preoperative CT examination is still difficult to distinguish PIP from other pulmonary diseases.^[19]^ Of the 25 cases undergoing preoperative CT examination in our hospital, 13 cases were considered as lung cancer or other malignant tumors, indicating that preoperative CT examination has some limitations in distinguishing PIP from lung cancer. Besides, PIP should also be differentiated from other benign and malignant lung diseases such as sclerosing alveolar cell tumor, pulmonary nodule, and tuberculoma. Therefore, if necessary, a lung puncture biopsy can be performed before the operation to confirm the diagnosis.

At present, the diagnosis of PIP still mainly depends on the pathological examination. Histologically, PIP is characterized by a variety of spindle cell proliferation in a myxoid to collagenous stroma with obvious inflammatory infiltrate composed primarily of lymphocytes and plasma cells, with occasional admixed neutrophils and eosinophils.^[20]^ In present study, we also found the collection of inflammatory mesenchymal cells (lymphocytes, plasma cells, and spindle cells). In terms of immunohistochemistry, spindle cells could express vimentin and smooth muscle actin. According to the literature, PIP is positive for smooth muscle actin in 80% to 90% of cases.^[21]^ In present study, only 8 patients underwent pathological immunohistochemical examination, of which 7 (87.5%) were positive for vimentin and 5 (62.5%) were positive for smooth muscle actin, which was slightly lower than that given in other reports.

Corticosteroid, radiation, and surgical therapy have been used for the treatment of PIP. For cases with inoperable, complicated heart-respiratory disease, unresectable lesions or recurrence, corticosteroid therapy has been proposed.^[22]^ Radiotherapy is usually used in patients with aggressive PIP or postoperative recurrence, or for patients at high surgical risk.^[23]^ By contrast, complete surgical resection is still the first choice for the treatment of PIP.^[24]^ It has been reported that patients with complete tumor resection have no recurrence, and the prognosis is generally good.^[25]^ For inflammatory myofibroblastic tumor limited to 1 organ, the recurrence rate varies by anatomical site, ranging from 1.8% for tumors confined to the lung to 8% for extrapulmonary lesions.^[17]^ In present study, complete tumor resection was obtained in all cases, and no obvious complications occurred after surgery. In addition, no recurrence or metastasis occurred during followed up, which was consistent with existing studies.

In conclusion, PIP is a rare tumor with unknown etiology, which has a variety of manifestations. Preoperative diagnosis is difficult to reach. The final diagnosis still depends on the pathological and immunohistochemical examination. Complete surgical resection is the main treatment at present, and the overall prognosis is good.

## Acknowledgments

None.

## Author contributions

Conception and design, D.W.; Data collection, H.C. and Q.F.; Data analysis and interpretation, H.C. and Y.S.; Drafting article, H.C.; Administrative support, D.W. All the authors have read and approved the final manuscript.

**Conceptualization:** Da-Li Wang.

**Data curation:** Heng-Chi Chen, Qiang Fu.

**Formal analysis:** Heng-Chi Chen, Yan Song.

**Methodology:** Heng-Chi Chen.

**Writing – review & editing:** Da-Li Wang.
